# Enhanced Shear Force Responsiveness of Epithelial Na^+^ Channel’s (ENaC) δ Subunit Following the Insertion of N-Glycosylation Motifs Relies on the Extracellular Matrix

**DOI:** 10.3390/ijms22052500

**Published:** 2021-03-02

**Authors:** Daniel Barth, Fenja Knoepp, Martin Fronius

**Affiliations:** 1 Institute of Physiology, Rheinisch-Westfälische Technische Hochschule Aachen, 52074 Aachen, Germany; dbarth@ukaachen.de; 2 Excellence-Cluster Cardio-Pulmonary Institute, Universities of Giessen and Marburg Lung Center, Member of the German Center for Lung Research, Justus-Liebig University Giessen, 35392 Giessen, Germany; Fenja.Knoepp@innere.med.uni-giessen.de; 3 Department of Physiology and HeartOtago, University of Otago, 9054 Dunedin, New Zealand

**Keywords:** shear force, extracellular matrix, N-glycan, epithelial Na^+^ channel, mechanotransduction, force-from-filament, tether

## Abstract

Members of the Degenerin/epithelial Na^+^ channel (ENaC) protein family and the extracellular cell matrix (ECM) form a mechanosensitive complex. A core feature of this complex are tethers, which connect the channel with the ECM, however, knowledge about the nature of these tethers is scarce. N-glycans of α ENaC were recently identified as potential tethers but whether N-glycans serve as a ubiquitous feature for mechanosensation processes remains unresolved. The purpose of this study was to reveal whether the addition of N-glycans to δ ENaC—which is less responsive to shear force (SF)—increases its SF-responsiveness and whether this relies on a linkage to the ECM. Therefore, N-glycosylation motifs were introduced via site-directed mutagenesis, the resulting proteins expressed with β and γ ENaC in *Xenopus* oocytes, and SF-activated currents measured by two-electrode voltage-clamp. The insertion of N-glycosylation motifs increases δ ENaC’s SF responsiveness. The inclusion of a glycosylated asparagine (N) at position 487 did increase the molecular mass and provided a channel whose SF response was abolished following ECM degradation via hyaluronidase. This indicates that the addition of N-glycans improves SF-responsiveness and that this effect relies on an intact ECM. These findings further support the role of N-glycans as tethers for mechanotransduction.

## 1. Introduction

The ability to sense and respond to mechanical cues is a common feature of all living organisms and the underlying process is referred to as mechanotransduction [1]. Common molecules for the initiation of mechanotransduction processes are, among others, mechanosensitive ion channels. One particular family of proteins forming mechanosensitive ion channels is the Degenerin/ENaC protein family. Proteins within this family were identified to be important for the ability to respond to touch in studies characterising mechanosensory neurons in *Caenorhabditis elegans*. These studies provided evidence that proteins encoded by the *deg-1* and *mec-4* genes in *C. elegans* were essential for touch sensation [2]. The discovery of a Degenerin homologue in vertebrates that encodes the alpha (α) subunit of the amiloride-sensitive epithelial sodium channel (ENaC) [3,4] revealed that *deg-1* and *mec-4* encode channel proteins that are involved in the transduction of mechanical force into electrical cellular signals [5]. Further studies by Chalfie and co-workers revealed that proteins that are part of the extracellular matrix (ECM) are also important for touch sensation in *C. elegans* [6,7]. This led to a model for mechanotransduction that comprises components of the ECM, ion channel proteins and intracellular cytoskeletal components [8]. A crucial component of this model is the existence of tethers. Tethers could provide a physical connection between the channel protein and intra-/extracellular matrices [9,10] to transduce deflections of the matrices into conformational changes of transmembrane (channel) proteins. This concept is one of the main principles for mechanotransduction known as “force-from-filament” [11].

Beside the force-from-filament principle, the force-from-lipids principle has been proposed [11,12]. Here the opening of the channel relies on membrane tension in response to force. Due to the interaction between the transmembrane parts of the channel protein and the surrounding phospholipids, membrane deformation will provide the energy for the conformational change of the channel to open its pore [12]. 

Although the mechanical gating of channels in accordance with the force-from-lipids principle is well characterised, experimental evidence for the force-from-filament principle remains scarce. Besides the studies in the *C. elegans* touch receptor, the existence of extracellular tethers was identified in stereocilia of hair cells. Here extracellular filamentous structures named “cross-links” [13] were identified that provided a physical connection between neighbouring stereocilia. In response to deflection of the cilia these cross-links, attached to channels, facilitate the mechanical opening of the channels as identified by changes in conductance as a response to displacement of stereocilia [14,15]. Although there is convincing evidence for the existence of extracellular tethers, their architecture remains largely unknown. 

In a recent study, the role of extracellular N-glycans for the ability of ENaC to respond to shear force was revealed [16]. The removal of certain asparagines within N-glycosylation motifs in its α subunit decreased the channel’s ability to respond to shear force. In addition, this study also provided evidence for the contribution of the ECM as an important component for the activation of ENaC in response to shear force because enzymatic degradation of the ECM had similar effects as the removal of asparagines. This strongly indicates that the function of the channel N-glycans and the ECM is interdependent as predicted by the force-from-filament principle. 

In the present study, the aim was to further support the interdependent activity between channel N-glycans and the ECM. Experiments were performed with the delta (δ) ENaC subunit. Channels containing this subunit are less shear force responsive in comparison with channels containing the α ENaC subunit [17]. A reason for this could be the lack of those asparagines that were identified to facilitate shear force responsiveness of α ENaC in conjunction with the ECM. Based on these characteristics it was decided to introduce N-glycosylation motifs into the δ ENaC subunit and reveal whether or not this is a suitable strategy to modify the channel’s ability to respond to shear force.

## 2. Results

**Figure 1:**
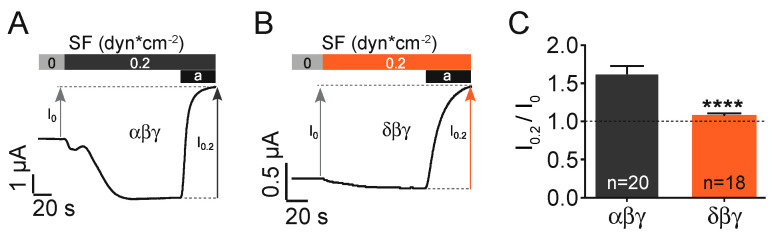
Shear force (SF) activation of αβγ and δβγ ENaC. Representative current traces (**A**,**B**) and statistics of the normalised shear force response of αβγ and δβγ ENaC (**C**). Amiloride (a) was used to determine ENaC-mediated currents in the absence (I_0_) and presence (I_0.2_) of shear force. The shear force response (I_0.2_/I_0_) of δβγ was smaller compared with αβγ (****, *p* < 0.0001, unpaired *t*-test; I_0_ indicated by the dashed line).

The expression of canonical αβγ ENaC in *Xenopus* oocytes resulted in the activation of an inward current in response to the activation of a flowing stream generating approx. 0.2 dynes*cm^−2^ of shear force. This current was inhibited by the application of the common ENaC inhibitor amiloride (10 µM, Figure 1A). In a series of identical experiments δβγ ENaC expressing oocytes were exposed to shear force (Figure 1B). Here the normalised shear force response (I_0.2_ dynes /I_0_ dynes) was considerably reduced in comparison with αβγ ENaC (Figure 1C). 

**Figure 2:**
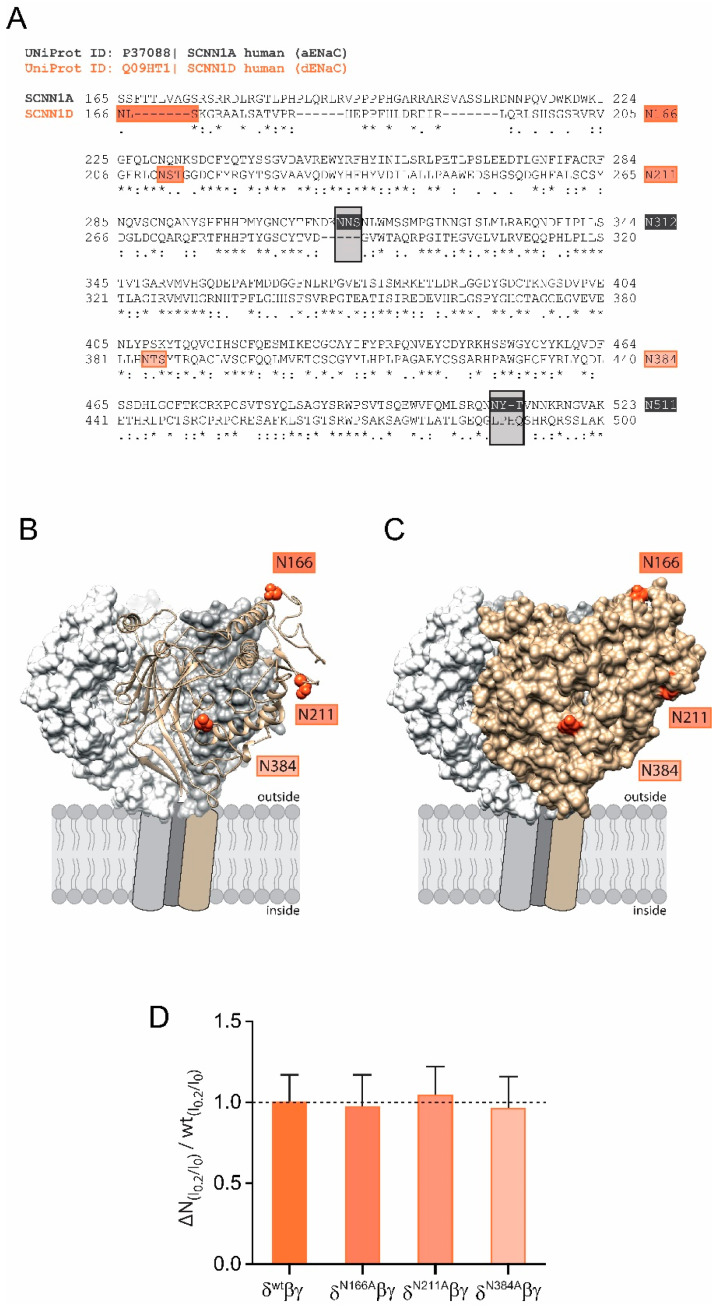
Shear force effect of δβγ ENaC is not mediated by intrinsic N-glycosylation sites. (**A**) Sequence alignment of the extracellular domains of human α (SCNN1A, grey) and δ (SCNN1D, orange) ENaC. N-glycosylation motifs of α ENaC that facilitate the shear force response are highlighted in black, whereas intrinsic N-glycosylation motifs of δ ENaC are coloured in orange. (**B**) Cartoon representation of the extracellular domain of human δ ENaC (highlighted in tan) together with wt β (grey) and γ subunit (white) that are shown as surface representation. The predicted localization of glycosylated asparagines 166, 211 and 384 within δ ENaC are highlighted as spheric molecules representation and coloured in orange. (**C**) Solvent accessible surface representation of δ ENaC indicates that the glycosylated asparagine residues are localised at exposed positions, likely to provide connections to the ECM. (**D**) Individual disruption of the three intrinsic N-glycosylation sites (∆N) in δ ENaC did not affect the shear force response (I_0.2_/I_0_ normalised for each construct) when compared with channels containing the wild type δ ENaC subunit (I_0.2_/I_0_ of δ^wt^βγ = 1 indicated by the dashed line, unpaired *t*-test).

The shear force response of αβγ ENaC relies on the presence of N-glycans attached to certain asparagines in the extracellular domain of the α subunit because the replacement of two asparagines (out of five) impaired ENaC’s ability to respond to shear force [16]. Interestingly, there are only three N-glycosylation sites in δ ENaC in comparison to five within the α subunit (N166, N211 and N384, Figure 2A). Comparative modelling of the human δ ENaC-subunit revealed that these asparagines are localised on the outer protein surface (Figure 2B,C) and could provide access to the ECM. However, individual replacement of these asparagines were found to not affect the shear force response when these subunits were co-expressed with β and γ ENaC in comparison with the shear force response of channels containing the wild type δ ENaC subunit (Figure 2D). The observations from Figure 1C and Figure 2D indicate two things: (1) the decreased shear force response of channels with wild type δ ENaC could be explained by the lacking asparagines; (2) the intrinsic asparagines of δ ENaC do not contribute to shear force sensing. It was therefore decided to introduce N-glycosylation motifs into the δ ENaC subunit aiming to assess how this is affecting the ability of the resulting channels to respond to shear force. 

**Figure 3:**
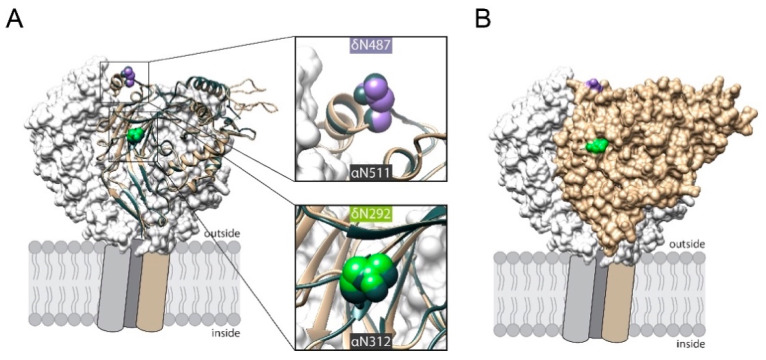
(**A**) Cartoon representation of human δ ENaC’s extracellular domain (highlighted in tan) that is aligned to human α ENaC, coloured in slate grey. Extracellular domains of wt β (grey) and γ subunit (white) are shown as surface representation. The predicted localization of the inserted asparagines 292 (green) and 487 (purple) are highlighted together with the corresponding asparagines in α ENaC (N312 and 511, slate grey). (**B**) Solvent accessible surface representation of δ ENaC indicates that the inserted asparagine residues are localised at exposed positions.

To address this question, three δ ENaC constructs were generated. One that had an N-glycosylation motif with an asparagine inserted at position 292 (N292) aiming to mimic N312 of the α subunit. One construct had an N-glycosylation motif inserted with an asparagine at position 487 (N487). N487 was considered to mimic N511 of α ENaC. Finally, one construct was generated that had both N-glycosylation motifs inserted (N292/N487). Comparative remodelling revealed that the localization of both inserted asparagines correspond to the respective N-glycosylated asparagines in α ENaC (Figure 3A) and are located at the outer surface of the protein (Figure 3B).

**Figure 4:**
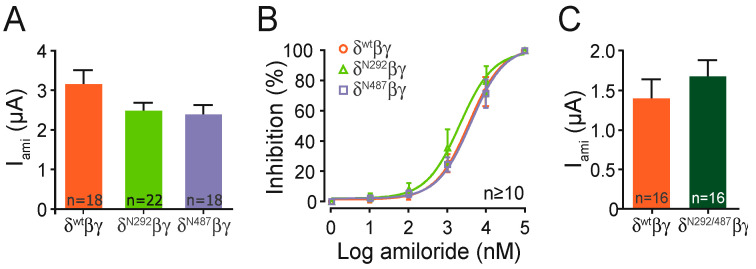
Insertion of N-glycosylation sites in δ ENaC does not impair channel function. (**A**) ENaC mediated amiloride-sensitive whole-cell currents (I_ami_) of δ^N292^βγ and δ^N487^βγ were comparable to δ^wt^βγ ENaC (one-way ANOVA with multiple comparisons). (**B**) Half-maximal amiloride concentrations for δN293 and δN487 constructs were also comparable to wt. (**C**) ENaC mediated currents (I_ami_) of δ^N292/N487^βγ channels were similar to wt channels (unpaired *t*-test).

Each construct containing a single glycosylation motif provided amiloride-sensitive whole-cell currents that were similar compared with the channel containing the wild type subunit (Figure 4A). In a separate set of experiments the construct including both motifs (N292/N487), also showed similar amiloride-sensitive currents when compared with the wild type channel (Figure 4C). This indicates that the basic ion channel function is not affected by the insertion of the N-glycosylation motifs. This is further supported by experiments determining the half-maximal amiloride concentration of the N293 and N487 constructs, which was also similar to the wild type (Figure 4B). 

**Figure 5:**
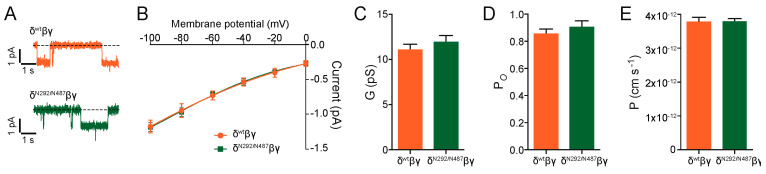
Single-channel properties of construct N292/N487 expressed with β and γ are comparable to wild type δβγ ENaC. (**A**) Patch-clamp recording in the cell-attached configuration. (**B**) The current-voltage relationship is similar between wt and N292/N487. Single-channel conductance (G, panel **C**), open probability (P*_O_*, panel **D**) and permeability (P, panel **E**) were comparable between δ^wt^βγ ENaC and δ^N292/N487^βγ (n ≥ 7, unpaired *t*-test).

Additional experiments were performed with the N292/N487 construct. Cell-attached single-channel recordings provided single-channel amplitudes and a conductance that was similar to the wild type channel (Figure 5). The single-channel open probability and single-channel permeability were unchanged. These findings indicate that the new channel constructs show similar basic channel function as the channels containing the wild type δ ENaC subunit. 

**Figure 6:**
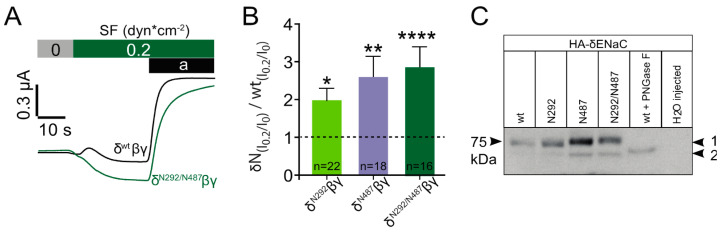
Insertion of N-glycosylation sites into δ ENaC increases the shear force effect. (**A**) Representative current traces of wild type δβγ ENaC and construct N292/N487 in response to shear force (SF). (**B**) The insertion of N-glycosylation motifs resulted in an increased shear force response (I_0.2_/I_0_) in comparison with wild type δβγ ENaC (*, *p* < 0.05, **, *p* < 0.001, ****, *p* < 0.0001; with respect to wt channel indicated by dashed line, unpaired *t*-test). (**C**) Immunoblotting of whole-cell lysates from oocytes expressing HA-tagged human δ ENaC (wt, N292, N487 and N292/N487) co-expressed with βγENaC. The shift in relative molecular weight is observed between the wt (~75 kDa) and construct N487 as well as the N292/N487 construct (arrow 1). The molecular weight of construct N292 was similar to the wt δ subunit. PNGase F resulted in the complete loss of the upper band (arrow 2) and water-injected oocytes served as control. Blot is representative for *n* = 4. Current traces of experiments from oocytes expressing δ^N292^βγ or δ^N487^βγ are depicted in Figure S1.

In a further set of experiments, each of these constructs was again co-expressed with β and γ ENaC and exposed to shear force. In these experiments, the application of shear force provided a stronger response with channels that contained either of the three constructs (Figure 6A,B). The largest increase in the normalised shear force response relative to corresponding wild type channels was observed with the N292/N487 construct, with N487 providing an almost similar response. Last but not least the migration pattern of each of the constructs was determined by immunoblotting. This experiment aimed to reveal potential differences in the molecular mass of the constructs as a consequence of the addition of N-glycans. In comparison with the wild type channel, a changed migration pattern was observed with N487 and N292/487 (Figure 6C). This indicates that these two constructs did have additional N-glycans that could contribute to the observed increased shear force activation. No difference in mass was observed with the N292 construct indicating that this construct was either not glycosylated or the glycan’s size was too small to be detected by the immunoblotting approach. 

**Figure 7:**
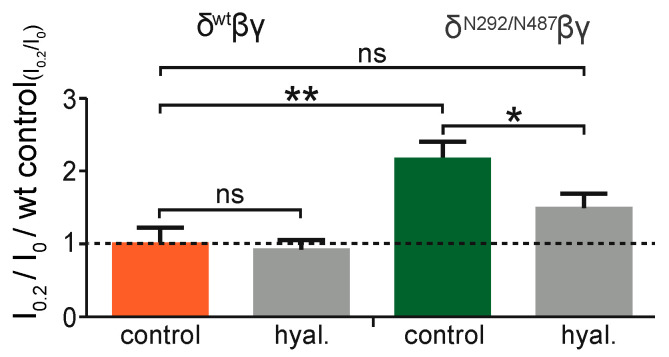
Degradation of hyaluronic acid of the ECM reduces the shear force response of channels containing the N292/N487 construct. Wild type δβγENaC and δ^N292/N487^βγ expressing oocytes were treated with hyaluronidase (hyal., grey bars). Hyaluronidase treatment had no effect on the shear force response of wild type δβγENaC, but reduced the shear force effect of δ^N292/N487^βγ (ns, *p* > 0.05; *, *p* < 0.05; **, *p* < 0.01; one-way ANOVA with multiple comparisons). Representative current traces of these experiments are depicted in Figure S2.

To reveal whether the activity of the added N-glycans relies on the ECM, experiments were performed with the N292/N487 construct. Here, channels were exposed to shear force in oocytes with an intact extracellular matrix in comparison to oocytes that were treated with hyaluronidase, an enzyme that degrades hyaluronic acid, a common component of extracellular matrices. This experiment was also repeated with channels containing the wild type δ ENaC subunit. As anticipated, the degradation of the ECM did not affect the shear force response in the wild type channel (Figure 7). In contrast to this, the N292/N487 construct did show an increased shear force response as expected in cells with intact ECM (Figure 6). However, this response was largely diminished in cells treated with hyaluronidase. 

## 3. Discussion

The present study revealed for the first time that the introduction of N-glycosylation motifs is a suitable strategy to facilitate the ion channel’s mechanical activation via a connection to the ECM. This is in agreement with a previous study providing evidence that both, channel N-glycans and the ECM, are important for shear force activation of ENaC [16]. However, the present study supports the findings from the Knoepp et al. study [16] but also indicates that the addition of glycosylation motifs is a suitable strategy to modify the ability of proteins to become more responsive to mechanical force (Figure 6). 

This was achieved by using δ ENaC, a subunit forming channels that are less responsive to shear force in comparison with channels containing the α ENaC subunit [17]. Further, the δ ENaC subunit exhibits a different N-glycosylation pattern in comparison with α ENaC. In contrast to α ENaC, δ ENaC does not have N-glycosylation motifs in the palm and knuckle domain. In α ENaC, asparagines at position 312 (palm domain) and 511 (knuckle domain) are important for the channel’s shear force response [16]. In the present study, the introduction of N-glycosylation motifs into δ ENaC, aiming to mimic N312 and N511 of α ENaC (Figure 3), resulted in channels that were more responsive to shear force in comparison to channels containing the wild type δ ENaC subunit. This was observed with the introduction of single N-glycosylation motifs (at position 292 or 487) and with a construct containing both. The increased shear force responsiveness is likely to be caused by modifying the ability to respond to shear force because basic channel functions (amiloride sensitive whole-cell current (Figure 4) and single-channel properties (Figure 5)) were not affected when compared with the wild type channel. For the two constructs containing the asparagine at position N487 the shear force response coincides with an increased molecular mass, likely due to the addition of N-glycans in the knuckle domain. Although the insertion of asparagine at position 292 did also result in an increased shear force response, the migration pattern of this construct was unchanged indicating that no N-glycan was added, or the attached N-glycan was too small to be detected. Difficulties to detect glycans is in agreement with previous reports suggesting that immunoblotting may not be sensitive enough to detect certain glycans [18,19]. Although being localised at exposed positions (Figure 2B,C), the intrinsic N-glycans of δ ENaC are not involved in the shear force responsiveness, because neither removal of N166, 211, or 384 of δ ENaC (Figure 2) changed the shear force response. This is in agreement with observations that N232, 293, 397 of α ENaC had also no effect on the channels ability to respond to shear force [16]. This implicates that for mechanical activation of ENaC, N-glycans need to be localised in certain positions. 

Intriguingly, the ability of the N292/487 construct to respond to shear force was dependent on the ECM. Enzymatic degradation of the hyaluronic acid component of the ECM did not change the shear force response of the wild type channel, indicating that the wt channel is not physically connected to the ECM. With the modified construct, an elevated shear response was observed and this response was largely abolished following degradation of the ECM by hyaluronidase. This provides strong support for the interdependent action of N-glycans and the ECM for shear force responsiveness. This is in agreement with the proposed force-from-filament principle of mechanotransduction and identifies extracellular N-glycans as components for the physical interaction between transmembrane proteins and the ECM. It opens up the possibility that N-glycans within certain structural motifs and with a certain (yet unknown) composition may serve as ubiquitous tethers, or at least as an important part of a tether. This is in agreement with a study from Virion and a co-workers [20]. Their study identified extracellular N-glycans to be essential for the mechanical activation of a G-protein coupled receptor. Here N-terminal N-glycans of the receptor were required for force transduction as a consequence of traction force generated from the specific binding of bacterial components [20,21].

The importance of glycans for protein function is supported by a growing awareness that they can serve as a “sugar code” providing a third alphabet [22], in addition to the genetic code (base sequence) and the protein code (amino acid sequence). This implicates that different glycan composition and structure due to the inclusion of different carbohydrates can have specific effects on protein function. With regard to mechanotransduction, there are three main reasons supporting the role of N-glycans as “prime” candidates for molecular tethers:

1. Attached to the extracellular domains of proteins they are extending from the protein surface into the extracellular space, placing them into a perfect position for making physical contacts between (neighbouring) molecules.
2. Glycans interact with lectins. Lectins are proteins that recognise and specifically bind carbohydrates [23]. Accordingly, lectins are suggested to be important for mediating ECM assembly, cell-cell and particularly cell-ECM interactions [22]. As important as the lectins are for mediating these interactions as important are the glycans. Thus, the interaction of ENaC glycans with lectins within the ECM is in agreement with the proposed force-from-filament concept. Lectins could transmit the movements of the ECM to the channel protein and induce a conformational change.
3. There is evidence for glycan-glycan interactions. This interaction between carbohydrates was discovered in sponges as an important way for cell-cell interactions to provide structural stability for the formation of multicellular three-dimensional structures [24,25].

Although it remains unknown whether ENaC N-glycans interact with lectins or other glycans there is growing support for the role of glycans as an enabler for the sensation and transduction of mechanical force [16,20]. It may also be taken into account that other structural domains may be involved in ENaC’s shear force response. Studies form the Kleyman group, which has initially identified ENaC’s ability to respond to shear force [26,27], have considerably improved our understanding of how various domains of ENaC proteins contribute to the gating process in response to shear force. This includes the pore region [28], the thumb domain [29] the transmembrane domain [17], the finger [30] and the wrist domain [31]. The shear force effect is also independent of the interaction with the membrane as proposed by the force-from-lipid concept [32]. This further emphasizes that shear force activation of ENaC is in agreement with the force-from-filament principle and the proposed role of N-glycans. A recent study also revealed that the replacement of multiple asparagines did affect channel maturation and trafficking but no obvious changes with regard to shear force activation were observed [33]. The reason for this discrepancy remains unknown but it might be speculated that the replacement of multiple glycosylation sites initiates compensatory effects that interfere with the function of the glycans that provide a connection to the ECM. It should also be acknowledged that the ability of ENaC to respond to shear force does not per se require N-glycans—they are however an important aspect to facilitate shear force responsiveness. 

In conclusion, results from this study indicate that N-glycans can serve as attachment points for the connection to the ECM to facilitate the transduction of mechanical force into the opening of a channel. The identification of N-glycans as tethers for mechanotransduction does provide a significant improvement for the understanding of mechanotransduction processes that were identified to rely on either the ECM (e.g., the endothelial glycocalyx being described as “shear sensor” [34]) or the activity of mechanosensitive channels. It may be speculated that the actual “mechanosensory complex” in some of these cases is a combination of the ECM (or glycocalyx) being physically connected to transmembrane proteins and their orchestrated activity is needed for mechanotransduction. N-glycans with their characteristics and known to facilitate molecular contacts are therefore likely to be of considerable importance for this physical interaction.

## 4. Materials and Methods

### 4.1. Oocyte Isolation and Preparation

All experiments performed within this study were approved by the Animal Ethics committee of the University of Otago (approvals #: 114/13 (2013), 83/16 (2016)). Female adult *Xenopus laevis* were kept in a Tecniplast (Sydney, Australia) housing system and fed once a week. For oocyte extraction, individual animals were anaesthetised in water from the housing system containing 1.3% MS-222 (tricaine methanesulfonate, Sigma-Aldrich, Auckland, New Zealand) buffered to a neutral pH. An incision was made through the abdominal skin and muscle layer and an ovarian lobe containing oocytes was removed and stored in culture oocyte Ringer’s solution (CulORi, containing in mM: 90 NaCl, 1 KCl, 2 CaCl_2_, 5 HEPES, 2.5 Na^+^-pyruvate, 0.06 penicillin, 0.02 streptomycin and in addition 50 μg/mL tetracycline, 100 μg/mL amikacin, 100 μg/mL ciprofloxacin, pH 7.4). Extracted ovarian lobes were incubated in collagenase (1.5 mg/mL dissolved in CulORi, Serva, Tauranga, New Zealand) for 90 min on a shaker followed by a 10-min incubation in Ca^2+^ free ORi (containing in mM: 90 NaCl, 5 HEPES, 1 KCl, 1 EGTA, pH 7.4) to separate cells and remove follicle cells. Fully developed and healthy-looking stage V–VI oocytes were collected and injected (Nanoject II Auto-Nanoliter Injector, Drummond Scientific, Broomall, PA, USA) with 0.24 ng total cRNA encoding α (NCBI# NM_001038.5) or δ (NCBI# NM_001130413.3) and β (NCBI# NM_000336.2) and γ (NCBI# NM_001039.3) ENaC in a ration of 1:1:1 for the relative amount per subunit. Cells were subsequently transferred into a 96 well plate containing low Na^+^-solution (containing in mM: 10 NaCl, 80 NMDG (N-methyl-D-glucamine), 1 KCl, 2 CaCl_2_, 5 HEPES, 2.5 Na^+^-pyruvate, 0.06 penicillin, 0.02 streptomycin and in addition 50 μg/mL tetracycline, 100 μg/mL amikacin, 100 μg/mL ciprofloxacin, pH 7.4). 

### 4.2. Molecular Biology

The pTNT vector (Promega, Madison, WI, USA) containing δ ENaC (with and without C-terminal HA-tag) was used as a template for site-directed mutagenesis (QuickChange Lightning site-directed mutagenesis kit, Life Technologies, Auckland, New Zealand). The primers used for the mutagenesis were designed according to the guidelines specified by the manufacturers manual and listed below. Mutated plasmids were transformed into competent DH5α *E. coli* cells followed by plasmid DNA purification according to the manufacturer’s protocol (QuickClean II Plasmid Miniprep Kit, GenScript, Piscataway, NJ, USA). The insertion of two glycosylation motifs occurred consecutively. cDNA was then sequenced to confirm the inclusion of glycosylation motifs. Finally, cDNA was linearised (BamHI, Roche, Auckland, New Zealand) and reverse transcribed in vitro using the SP6 mMESSAGE mMACHINE Kit (Ambion, Austin, TX, USA) according to the manufacturer’s instructions and stored at −80 °C. 

Primers used for site-directed mutagenesis are listed in Table 1 with altered nucleotide sequence highlighted in bold. For construct N292, the three amino acids NNS were inserted between amino acids 291 and 292 increasing the total construct length by three amino acids. For construct N487, amino acids LPH (position 487–489) were replaced by NYT. 

### 4.3. Proposed Structures of Human δENaC by Comparative Modelling 

The full-length extracellular domain of human ENaC at 3 Å (PDB: 6WTH [35]) was chosen as a template for creating predicted 3D models of δ^wt^ and δ^N292/487^ ENaC. Therefore, the amino acid sequence of the extracellular domain of human δ ENaC (UniProt identifier Q09HT1) was used as input and aligned to 6WTH_A (human α ENaC) using UCSF Chimera 1.15rc [36] with the BLOSUM62 scoring matrix. For δ^N292/487^ ENaC, the respective N-glycosylation motifs (as specified in Section 4.2) were inserted manually into the δ^wt^ ENaC sequence at the appropriate positions and subsequently used as input for homology modelling. 5 different models for each subunit (δ^wt^ and δ^N292/487^ ENaC) were generated via implemented comparative modelling function (http://www.salilab.org/modeller/ (accessed on 23 February 2021) [37]). The model with the lowest normalised Discrete Optimized Protein Energy (zDOPE)— score was selected as the best model and shown together with wt β and γ ENaC (PDB: 6WTH_B and 6WTH_C). To indicate the putative location of selected asparagines relative to the 3D space of the extracellular domain, the ‘surface’ function was used to visualise the solvent-accessible surface area.

### 4.4. Western Blotting

δβγENaC (δ-ENaC included C-terminal HA-tag) transfected oocytes were homogenised after 48 h incubation in lysis buffer (containing in mM: 10 HEPES, 83 NaCl, 1 MgCl_2_, 1 Triton X-100, complete mini EDTA-free protease inhibitor cocktail (Merck, Auckland, New Zealand) and protein concentrations determined using the DC^TM^ Protein Assay kit (Bio-Rad, Hercules, CA, USA) according to the manufacturer’s instructions. Protein lysates (20 µg) were either directly subjected to reducing SDS-PAGE and Western-blot analysis or following the treatment with PNGase F (New England Biolabs, Ipswich, MA, USA) according to supplier’s protocol to deglycosylate the samples. Denaturation of the samples was performed using 5 × Laemmli sample buffer (containing in %: 10 Tris, 5 SDS, 25 Glycerol, 0.8 bromophenol blue, 5 b-mercaptoethanol) and incubated for 10 min at 100 °C. Samples were then loaded onto mini protein gels (Invitrogen, Auckland, New Zealand) and run at 120 V for 2 hrs using a Mini-Vertical system (Hoefer, Holliston, MA, USA). Protein samples were transferred to PVDF membranes at 25 V for 30 min using a Trans-Blot Turbo Transfer Starter System (Bio-Rad). The membranes were then blocked for 1 hr in TBS-Tween (containing in mM: 50 Tris, 150 NaCl and 0.1 % Tween) with 5 % milk, followed by overnight incubation at 4 °C in TBS-T containing the primary antibody (Anti-Ha, 1:1000, polyclonal, rabbit, Sigma-Aldrich). Membranes were then incubated with horseradish peroxidase conjugated secondary antibody (Anti-rabbit, 1:5000 in TBS-T, polyclonal, goat, GE Healthcare, Chicago, IL, USA) for 2 h at room temperature and subsequently subjected to chemiluminescent detection (Amersham ECL prime, Sigma-Aldrich) and visualised via an X-ray film (Radiographic Supplies, Christchurch, New Zealand). 

### 4.5. Electrophysiological Recordings

#### 4.5.1. Two-Electrode-Voltage-Clamp (TEVC) Recordings 

Transmembrane currents were recorded using a TURBO TEC-05 amplifier (NPI, Tamm, Germany), digitised via a PowerLab 4/35 (ADInstruments, Dunedin, New Zealand) and recorded through LabChart (ADInstruments). Oocytes were placed in a custom-made shear force chamber containing oocyte Ringer’s solution (containing in mM: 90 NaCl, 1 KCl, 2 CaCl_2_, 5 HEPES, pH 7.4). The chamber was connected to a modified perfusion system (ALA Scientific Instruments, Farmingdale, New York, USA) that included a pressure gauge and was connected to pressurised air. This system allowed accurate adjustment of perfusion rates and consistent application of shear force onto an oocyte that was placed in the centre of the flow-channel of the chamber. The microelectrodes were chloride silver wires placed in a borosilicate glass capillary (2–4 MΩ, Hilgenberg, Malsfeld, Germany) filled with 1 M KCl. Once electrodes were impaled, the membrane potential of the oocytes was clamped to –60 mV. Shear force application occurred by activation of the chamber perfusion with a flow rate of 2.4 mL/min that corresponded to a shear force rate of about 0.2 dyn*cm^–2^ on the surface of the oocyte. Shear force rates were calculated as described previously [38]. 

#### 4.5.2. Single-Channel Recordings

Cell-attached recordings on oocytes were performed using an Axopatch 200B amplifier (Molecular Devices, San Jose, CA, USA) and digitised via a Digidata 1440A (Molecular Devices). Prior to data acquisition, currents were filtered with 100 Hz using a low-pass filter (900 CT Tunable Active Filter, Frequency Devices, Ottawa, IL, USA) and a Humbug 50 Hz noise eliminator (Quest Scientific, North Vancouver, BC, Canada). Data was acquired and digitised at 2 kHz and analysed with Axon Clampex 10.4 and Clampfit 10.4 (Molecular Devices). Patch Pipettes were made of borosilicate glass (Hilgenberg) and had tip resistances of 3 to 10 MΩ. Microelectrodes were filled with the extracellular solution (containing in mM: 90 NaCl, 1 KCl, 2 CaCl_2_, 5 HEPES, pH 7.4). A high K^+^ bath solution (containing in mM: 145 KCl, 1.8 CaCl_2_, 2 MgCl_2_, 5.5 glucose, 10 HEPES, pH 7.2) was used to keep the endogenous membrane potential close to 0 mV. Prior to the recordings, oocytes were devitellinised by osmotic shrinkage and forceps using CulORi including 10% Mannitol. Single-channel currents were recorded between −100 and 0 mV to obtain single-channel amplitudes for the calculation of the channel’s conductance and permeability. The open probability was recorded in separate recordings at −100 mV over a period of approximately 10 min. A detailed description of how the single-channel parameter were analysed can be observed in a previous publication [38]. 

### 4.6. Degradation of the Extracellular Matrix (ECM)

Degradation of the ECM of oocytes was performed by incubating the cells with hyaluronidase. In short, prior to the TEVC experiment, oocytes were incubated in 300 U/mL hyaluronidase (OmniPur Merck, North Shore City, New Zealand) dissolved in CulORi at pH 6 for 2 min at room temperature. Control oocytes were incubated in CulORi at pH 6 for 2 min at room temperature. Following the incubation, oocytes were immediately used for two-electrode voltage-clamp experiments. 

### 4.7. Data Analyses and Statistics 

Data are expressed as mean ± standard error of the mean (SEM). The numbers of experiments are presented as n and data for each experiment were obtained using oocytes from at least two different animals. Electrophysiological data were analysed using GraphPad Prism 7. Statistical comparisons were performed using unpaired *t*-test, paired Student’s *t*-test or multiple comparisons one-way ANOVA and are specified within the corresponding figure legends. Statistical differences were indicated as following ns = *p* ≥ 0.05; * = *p* < 0.05; ** = *p* < 0.01; *** = *p* < 0.001; **** = *p* < 0.0001.

## Figures and Tables

**Table 1:** Nucleotide sequence of primers used for the site-directed mutagenesis to insert N-glycosylation motifs. Altered/modified sequences are bold.

Construct	Orientation	Sequence (5′-3′)
N292	Forward	GCTACACGGTCGAT**AATAATTCA**GGCGTCTGGACAGC
	Reverse	GCTGTCCAGACGCC**TGAATTATT**ATCGACCGTGTAGC
N487	Forward	CTAGGTGAACAGGGG**AATTACACT**CAGAGCCACAGACAG
	Reverse	CTGTCTGTGGCTCTG**AGTGTAATT**CCCCTGTTCACCTAG

## Data Availability

The data presented in this study are available on request from the corresponding author.

## Supplementary Materials

The following are available online at https://www.mdpi.com/1422-0067/22/5/2500/s1.

## References

[1] Hamill O.P., Martinac B. (2001). Molecular basis of mechanotransduction in living cells. Physiol. Rev..

[2] Driscoll M., Chalfie M. (1991). The mec-4 gene is a member of a family of Caenorhabditis elegans genes that can mutate to induce neuronal degeneration. Nature.

[3] Canessa C.M., Horisberger J.D., Rossier B.C. (1993). Epithelial sodium channel related to proteins involved in neurodegeneration. Nature.

[4] Chalfie M., Driscoll M., Huang M. (1993). Degenerin similarities. Nature.

[5] Chalfie M. (1993). Touch receptor development and function in Caenorhabditis elegans. J. Neurobiol..

[6] Du H., Gu G., William C.M., Chalfie M. (1996). Extracellular proteins needed for C. elegans mechanosensation. Neuron.

[7] Emtage L., Gu G., Hartwieg E., Chalfie M. (2004). Extracellular proteins organize the mechanosensory channel complex in C. elegans touch receptor neurons. Neuron.

[8] Bounoutas A., Chalfie M. (2007). Touch sensitivity in Caenorhabditis elegans. Pflugers Arch..

[9] Chalfie M. (2009). Neurosensory mechanotransduction. Nat. Rev. Mol. Cell Biol..

[10] Cox C.D., Bavi N., Martinac B. (2019). Biophysical Principles of Ion-Channel-Mediated Mechanosensory Transduction. Cell Rep..

[11] Katta S., Krieg M., Goodman M.B. (2015). Feeling Force: Physical and Physiological Principles Enabling Sensory Mechanotransduction. Annu. Rev. Cell Dev. Biol..

[12] Cox C.D., Bavi N., Martinac B. (2018). Bacterial Mechanosensors. Annu. Rev. Physiol..

[13] Pickles J.O., Comis S.D., Osborne M.P. (1984). Cross-links between stereocilia in the guinea pig organ of Corti, and their possible relation to sensory transduction. Hear Res..

[14] Corey D.P., Hudspeth A.J. (1979). Response latency of vertebrate hair cells. Biophys. J..

[15] Corey D.P., Hudspeth A.J. (1983). Kinetics of the receptor current in bullfrog saccular hair cells. J. Neurosci..

[16] Knoepp F., Ashley Z., Barth D., Baldin J.P., Jennings M., Kazantseva M., Saw E.L., Katare R., Alvarez de la Rosa D., Weissmann N. (2020). Shear force sensing of epithelial Na+ channel (ENaC) relies on N-glycosylated asparagines in the palm and knuckle domains of αENaC. Proc. Natl. Acad. Sci. USA.

[17] Abi-Antoun T., Shi S., Tolino L.A., Kleyman T.R., Carattino M.D. (2011). Second transmembrane domain modulates epithelial sodium channel gating in response to shear stress. Am. J. Physiol. Renal Physiol..

[18] Maley F., Trimble R.B., Tarentino A.L., Plummer T.H.J. (1989). Characterization of glycoproteins and their associated oligosaccharides through the use of endoglycosidases. Anal. Biochem..

[19] Morelle W., Michalski J.C. (2007). Analysis of protein glycosylation by mass spectrometry. Nat. Protoc..

[20] Virion Z., Doly S., Saha K., Lambert M., Guillonneau F., Bied C., Duke R.M., Rudd P.M., Robbe-Masselot C., Nassif X. (2019). Sialic acid mediated mechanical activation of β_2_ adrenergic receptors by bacterial pili. Nat. Commun..

[21] Marullo S., Doly S., Saha K., Enslen H., Scott M.G.H., Coureuil M. (2020). Mechanical GPCR Activation by Traction Forces Exerted on Receptor N-Glycans. ACS Pharmacol. Transl. Sci..

[22] Kaltner H., Abad-Rodríguez J., Corfield A.P., Kopitz J., Gabius H.J. (2019). The sugar code: Letters and vocabulary, writers, editors and readers and biosignificance of functional glycan-lectin pairing. Biochem. J..

[23] Weis W.I., Drickamer K. (1996). Structural basis of lectin-carbohydrate recognition. Annu. Rev. Biochem..

[24] Bucior I., Scheuring S., Engel A., Burger M.M. (2004). Carbohydrate-carbohydrate interaction provides adhesion force and specificity for cellular recognition. J. Cell Biol..

[25] Popescu O., Checiu I., Gherghel P., Simon Z., Misevic G.N. (2003). Quantitative and qualitative approach of glycan-glycan interactions in marine sponges. Biochimie.

[26] Satlin L.M., Sheng S., Woda C.B., Kleyman T.R. (2001). Epithelial Na(+) channels are regulated by flow. Am. J. Physiol. Renal Physiol..

[27] Carattino M.D., Sheng S., Kleyman T.R. (2004). Epithelial Na+ channels are activated by laminar shear stress. J. Biol. Chem..

[28] Carattino M.D., Sheng S., Kleyman T.R. (2005). Mutations in the pore region modify epithelial sodium channel gating by shear stress. J. Biol. Chem..

[29] Shi S., Ghosh D.D., Okumura S., Carattino M.D., Kashlan O.B., Sheng S., Kleyman T.R. (2011). Base of the thumb domain modulates epithelial sodium channel gating. J. Biol. Chem..

[30] Shi S., Blobner B.M., Kashlan O.B., Kleyman T.R. (2012). Extracellular finger domain modulates the response of the epithelial sodium channel to shear stress. J. Biol. Chem..

[31] Shi S., Carattino M.D., Kleyman T.R. (2012). Role of the wrist domain in the response of the epithelial sodium channel to external stimuli. J. Biol. Chem..

[32] Carattino M.D., Liu W., Hill W.G., Satlin L.M., Kleyman T.R. (2007). Lack of a role of membrane-protein interactions in flow-dependent activation of ENaC. Am. J. Physiol. Renal Physiol..

[33] Kashlan O.B., Kinlough C.L., Myerburg M.M., Shi S., Chen J., Blobner B.M., Buck T.M., Brodsky J.L., Hughey R.P., Kleyman T.R. (2018). N-linked glycans are required on epithelial Na^+^ channel subunits for maturation and surface expression. Am. J. Physiol. Renal Physiol..

[34] Tarbell J.M., Ebong E.E. (2008). The endothelial glycocalyx: A mechano-sensor and -transducer. Sci. Signal..

[35] Noreng S., Posert R., Bharadwaj A., Houser A., Baconguis I. (2020). Molecular principles of assembly, activation, and inhibition in epithelial sodium channel. eLife.

[36] Pettersen E.F., Goddard T.D., Huang C.C., Couch G.S., Greenblatt D.M., Meng E.C., Ferrin T.E. (2004). UCSF Chimera—A visualization system for exploratory research and analysis. J. Comput. Chem..

[37] Sali A., Blundell T.L. (1993). Comparative protein modelling by satisfaction of spatial restraints. J. Mol. Biol..

[38] Althaus M., Bogdan R., Clauss W.G., Fronius M. (2007). Mechano-sensitivity of epithelial sodium channels (ENaCs): Laminar shear stress increases ion channel open probability. FASEB J..

