# Supplementary material for: Enhanced Shear Force Responsiveness of Epithelial Na+ Channel’s (ENaC) δ Subunit Following the Insertion of N-Glycosylation Motifs Relies on the Extracellular Matrix
Source: Int J Mol Sci. 2021 Mar 2;22(5):2500. doi: 10.3390/ijms22052500 (PMC7958617; doi:10.3390/ijms22052500)
Supplement: Supplementary file 1 [file ijms-22-02500-s001.pdf]

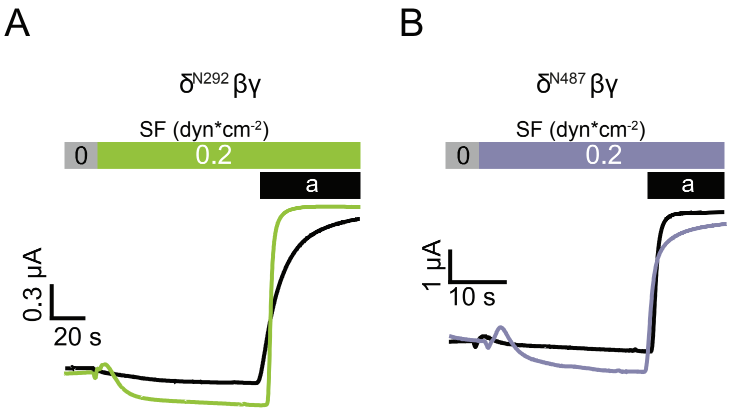

Supplemental Figure S1: Representative current traces for experiments summarized in Figure 6B. Delta ENaC constructs with single N-glycosylation sites inserted into the extracellular loop were more responsive to shear force compared with channels containing the wild type subunit (black traces).

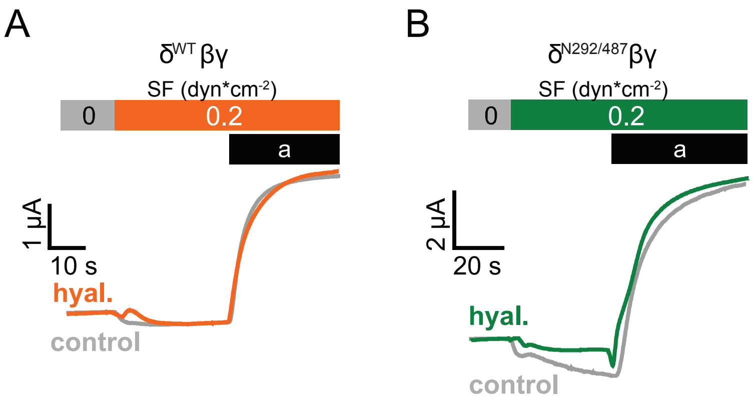

Supplemental Figure S2: Representative current traces for experiments summarized in Figure 7. Wild type or the construct containing two glycosylation sites were exposed to shear force in the absence (control) or following incubation with hyaluronidase (hyal.). The hyaluronidase treatment did reduce the shear force effect of the modified construct but had no effect on channels containing the wild type subunit.
